# *Stratiformator vulcanicus* gen. nov., sp. nov., a marine member of the family *Planctomycetaceae* isolated from a red biofilm in the Tyrrhenian Sea close to the volcanic island Panarea

**DOI:** 10.1007/s10482-023-01860-x

**Published:** 2023-08-16

**Authors:** Gaurav Kumar, Nicolai Kallscheuer, Mareike Jogler, Sandra Wiegand, Anja Heuer, Christian Boedeker, Manfred Rohde, Christian Jogler

**Affiliations:** 1 Department of Microbial Interactions, Friedrich Schiller University, Jena, Germany; 2 Cluster of Excellence Balance of the Microverse, Friedrich Schiller University, Jena, Germany; 3 Institute for Biological Interfaces 5, Karlsruhe Institute of Technology, Eggenstein-Leopoldshafen, Germany; 4 Leibniz Institute DSMZ, Brunswick, Germany; 5 Central Facility for Microscopy, Helmholtz Centre for Infection Research, Brunswick, Germany

**Keywords:** *Planctomycetota*, Asymmetric cell division, Polar budding, Marine bacteria, Mediterranean Sea, Biofilm, Hydrothermal vent system

## Abstract

A novel planctomycetal strain, designated Pan189^T^, was isolated from biofilm material sampled close to Panarea Island in the Tyrrhenian Sea. Cells of strain Pan189^T^ are round grain rice-shaped, form pink colonies and display typical planctomycetal characteristics including asymmetric cell division through polar budding and presence of crateriform structures. Cells bear a stalk opposite to the division pole and fimbriae cover the cell surface. Strain Pan189^T^ has a mesophilic (optimum at 24 °C) and neutrophilic (optimum at pH 7.5) growth profile, is aerobic and heterotrophic. Under laboratory-scale cultivation conditions, it reached a generation time of 102 h (µ_max_ = 0.0068 h^−1^), which places the strain among the slowest growing members of the phylum *Planctomycetota* characterized so far. The genome size of the strain is with 5.23 Mb at the lower limit among the family *Planctomycetaceae* (5.1–8.9 Mb). Phylogenetically, the strain represents a novel genus and species in the family *Planctomycetaceae*, order *Planctomycetales*, class *Planctomycetia*. We propose the name *Stratiformator vulcanicus* gen. nov., sp. nov. for the novel taxon, that is represented by the type strain Pan189^T^ (= DSM 101711^ T^ = CECT 30699^ T^).

## Introduction

Since the discovery of the first member in the year 1924, the phylogeny of planctomycetes was subject of controversy. Members of the later introduced phylum were first recognised as eukaryotes due to their morphology resembling fungi (Gimesi 1924). However, this misconception was revised, and the members were acknowledged as bacteria in 1972 (Hirsch 1972). The phylum *Planctomycetota*, along with other bacterial phyla like *Verrucomicrobiota, Kiritimatiellota*, *Lentisphaerota,* and *Chlamydiota*, form the PVC superphylum, whose members have biotechnological and/or medical relevance (Wagner and Horn 2006). In particular members of the class *Planctomycetia*, the class within the phylum *Planctomycetota* with the currently highest number of validly described species, show additional peculiarities regarding their cell biology (Vitorino and Lage 2022). One example is the use of an unusual form of asymmetric cell division (referred to as “budding”) that is not understood in detail (Rivas-Marin et al. 2020a). Recently, it was reported that some members even show more uncommon forms of lateral instead of polar cell division (Kumar et al. 2021; Wiegand et al. 2020b; Vitorino et al. 2020). The other two classes of the phylum, *Candidatus* Brocadiae and *Phycisphaerae* usually divide by binary fission (Kartal et al. 2013; Fukunaga et al. 2009). Independent of the observed mode of cell division, *ftsZ*, the gene encoding the otherwise essential canonical bacterial tubulin-homolog is absent in all analysed planctomycetal genomes and proteins that could have a related function during cell division have not yet been identified (Jogler et al. 2012; Wiegand et al. 2020b). Members of the phylum *Planctomycetota* often show a dimorphic lifestyle. Young cells are planktonic swimmers bearing flagella that subsequently attach to a surface through their holdfast structure and become sessile stalked mother cells. The stalked cells start reproducing to yield another generation of daughter cells (Jogler et al. 2011; Wiegand et al. 2018). From a genomic perspective, members of the phylum are noteworthy as their average genome size is 7.9 Mb; with *Phycisphaera mikurensis* having the currently smallest genome (3.9 Mb) and *Fimbriiglobus ruber* belonging to the family *Gemmataceae* having the currently largest (12.4 Mb) (Fukunaga et al. 2009; Ravin et al. 2018). Their large genomes and presence of ‘giant genes’ (with an open reading frame > 15 kb) makes them suitable sources for bioprospection studies targeting the discovery of novel secondary metabolites (Kallscheuer and Jogler 2021). In addition to enzymes belonging to the classes of non-ribosomal peptide synthetases and type I polyketide synthases, giant genes often also code for large extracellular adhesion proteins, many of which are potentially required during the suspected complex interaction of members of the phylum with phototrophs (Lage and Bondoso 2014; Reva and Tümmler 2008). The interactions are probably orchestrated by various bioinformatically predicted biosynthetic gene clusters (BGCs) in their genomes and experimentally evidenced secondary metabolites like, for example, the plant toxin 3,5-dibromo-*p*-anisic acid and biosurfactant compounds named stieleriacines (Kallscheuer et al. 2020a; Panter et al. 2019; Sandargo et al. 2020).

Within *Planctomycetia* falls the order *Planctomycetales* with the currently sole family *Planctomycetaceae.* The latter is the currently second largest family within the different orders of the class *Planctomycetia* and is currently constituted by fifteen genera: *Alienimonas, Calycomorphotria, Caulifigura, Fuerstiella, Gimesia, Maioricimonas, Planctomicrobium, Planctomyces, Planctopirus, Polystyrenella, Rubinisphaera, Schlesneria, Symmachiella, Thalassoglobus,* and *Thalassoroseus* (Boersma et al. 2020; Kallscheuer et al. 2020c; Kohn et al. 2016; Kulichevskaya et al. 2015, 2007; Kumar et al. 2022; Peeters et al. 2020; Rivas-Marin et al. 2020b; Salbreiter et al. 2020; Scheuner et al. 2014; Schubert et al. 2020). Despite the moderate number of species, the family *Planctomycetaceae* is still understudied as eight genera contain only a single species.

To expand the current collection of planctomycetes, we explored the uncultivated diversity close to the Lipari Islands in the Tyrrhenian Sea. The sampling site was located close to Panarea, the smallest of the volcanic islands north of Sicily, southern Italy. Several hydrothermal vents are in proximity to the island and the microorganisms of this ecosystem represent the basis of the hydrothermal system food web responsible for the transformation of inorganic compounds released from vent emissions (Jannasch and Mottl 1985; Karl 1995). Microorganisms of different phyla including *Planctomycetota* live at the shallow hydrothermal system near volcanic islands. Several cultivation-independent studies showed a large bacterial diversity in this area and our research group has already isolated novel planctomycetal strains from this spot (Kallscheuer et al. 2020b; Rensink et al. 2020; Wiegand et al. 2020a). Here, we describe another novel strain that was isolated close to the volcanic island Panarea, and characterize its morphology, physiology, phylogeny, and genome.

## Material and methods

### Habitat and isolation

Strain Pan189^T^ was isolated in a hydrothermal area (sampling site 38.5568 N, 15.1097 E, date: 9th September 2013) ca. 7 km southeast of the island Panarea in the north of Sicily, Italy. The sampled material was a red biofilm that was scraped from a rock located under the water surface. In the laboratory, the biofilm was transferred to sterile seawater containing 20 mg/L cycloheximide to prevent fungal growth and streaked on M1H NAG ASW solid medium containing 8 g/L gelrite additionally supplemented with 20 mg/L cycloheximide, 1000 mg/ L streptomycin and 200 mg/L ampicillin. M1H NAG ASW medium contained (g/L in double distilled water, pH 7.5): peptone, 0.25; yeast extract, 0.25; 4-(2-hydroxyethyl)-1-piperazineethanesulfonic acid) (HEPES), 2.38 g; 250 mL artificial seawater (ASW) and 20 mL sterile-filtered Hutner’s basal salt solution. The pH was adjusted to 7.5 using 5 M KOH and the medium was filled up to a volume of 973 mL with double distilled water. The solution was autoclaved for 20 min at 121 °C. After cooling, the following solutions were added aseptically: 1 mL of 25% (w/v) glucose, 5 mL vitamin solution, 1 mL trace element solution and 20 mL of a stock solution with 50 g/L *N-*acetyl glucosamine (NAG). ASW contained (g/L in double distilled water): NaCl, 46.94; Na_2_SO_4_, 7.84; MgCl_2_ × 6 H_2_O, 21.28; CaCl_2_ × 2 H_2_O, 2.86; NaHCO_3_, 0.384; KCl, 1.384; KBr, 0.192; H_3_BO_3_, 0.052; SrCl_2_ × 6 H_2_O, 0.08; and NaF, 0.006. Hutner’s basal salt solution contained (in g/L): nitrilotriacetic acid, 10; MgSO_4_ × 7 H_2_O, 30; CaCl_2_ × 2 H_2_O, 3.5; (NH_4_)_6_MoO_7_O_24_ × 4 H_2_O, 0.01; FeSO_4_ × 7 H_2_O, 0.1; and metal salt solution, 50 mL. The metal salt solution contained (in g/L): Na-EDTA, 0.25; ZnSO_4_ × 7 H_2_O, 1.1; FeSO_4_ × 7 H_2_O, 0.5; MnSO_4_ × H_2_O, 0.15; CuSO_4_ × 5 H_2_O, 0.04; Co(NO_3_)_2_ × 6 H_2_O, 0.025; Na_2_B_4_O_7_ × 10 H_2_O, 0.018. The vitamin solution contained (in mg/L): vitamin B_12_, 0.2; biotin, 4; thiamine-HCl × 2 H_2_O, 10; calcium pantothenate, 10; folic acid, 4; riboflavin, 10; nicotinamide, 10; *p*-aminobenzoic acid, 10; pyridoxine × HCl, 20. The trace element solution contained (in mg/L): Na-nitrilotriacetate, 1500; MnSO_4_ × H_2_O, 500; FeSO_4_ × 7 H_2_O, 100; Co(NO_3_)_2_ × 6 H_2_O, 100; ZnCl_2_, 100; NiCl_2_ × 6 H_2_O, 50; H_2_SeO_3_, 50; CuSO_4_ × 5 H_2_O, 10; AlK(SO_4_)_2_ × 12 H_2_O, 10; H_3_BO_3_, 10; NaMoO_4_ × 2 H_2_O, 10; and Na_2_WO_4_ × 2 H_2_O, 10. All solutions that were added after the autoclavation step were filter-sterilised and stored at 4 °C in the dark. The plate with the streaked biofilm material was incubated at 20 °C for twelve weeks. A pink colony from this plate was further purified through repeated streaking on an agar plate containing the same medium. The axenic culture was maintained in liquid M1H NAG ASW medium. The initial amplification and sequencing of the 16S rRNA gene was performed as previously described (Rast et al. 2017).

### Genome sequencing and annotation

Sequencing of the genome of strain Pan189^T^ with Illumina MiSeq is part of a previous study and details can be found in the respective publication (Wiegand et al. 2020b). The genome sequencing yielded a closed genome with 5,228,745 bp that is deposited at GenBank under accession number CP036268.1. The 16S rRNA gene sequence can be found under acc. no. MK554509.

### Analysis of genome-encoded features

The pangenome of the analysed strains was constructed with the pangenomics workflow of anvi’o v. 7.1 (Eren et al. 2021). The “Estimate Metabolism” function of the same tool was used for the analysis of genome-encoded primary metabolic functions. The numbers of putative carbohydrate-active enzymes (CAZymes) were obtained from the genome annotation provided by eggnog-mapper v.2.1.10 (Cantalapiedra et al. 2021). The in silico prediction of biosynthetic gene clusters (BGCs) putatively involved in the biosynthesis of secondary metabolites was carried out using antiSMASH 7.0 (Blin et al. 2021). The prediction was run with relaxed strictness, antiSMASH beta features and all extra features activated.

### Phylogenetic analysis

The 16S rRNA gene sequence of strain Pan189^T^ was extracted from the annotated genome and the identification of the closest neighbours of the novel isolate was performed using NCBI BLAST (Johnson et al. 2008). The 16S rRNA gene sequences of strain Pan189^T^ and all characterized members of the phylum were aligned with ClustalW (Thompson et al. 2003). The alignment was used to calculate a 16S rRNA similarity matrix with TaxonDC (Tarlachkov and Starodumova 2017). The 16S rRNA gene sequence-based maximum likelihood phylogenetic tree was calculated from the same alignment with FastTree 2.1 (Price et al. 2010) employing the GTR + CAT model and 1000 bootstraps replications. Three 16S rRNA genes of PVC superphylum strains, outside of the phylum *Planctomycetota*, namely *Opitutus terrae* (NCBI acc. no. AJ229235), *Kiritimatiella glycovorans* (acc. no. NR_146840) and *Lentisphaera araneosa* (acc. no. NR_027571), were used as outgroup. For the multi-locus sequence analysis (MLSA), the unique single-copy core genome of all analysed genomes was determined with proteinortho5 (Lechner et al. 2011) with the ‘selfblast’ option enabled. The protein sequences of the resulting orthologous groups were aligned using MUSCLE v.3.8.31 (Edgar 2004). After clipping, partially aligned *C*- and *N*-terminal regions and poorly aligned internal regions were filtered using Gblocks (Castresana 2000). The final alignment was concatenated and clustered using the maximum likelihood method implemented by RAxML (Stamatakis 2014) with the ‘rapid bootstrap’ method and 500 bootstrap replicates. The genomes of three strains from the family *Pirellulaceae*, namely *Pirellula staleyi* DSM 6068^ T^ (GenBank acc. no. GCA_000025185.1), *Blastopirellula marina* DSM 3645^ T^ (acc. no. GCA_000153105.1), and *Rhodopirellula baltica* SH1^T^ (acc. no. GCA_000196115.1), were used as outgroup. The average nucleotide identity (ANI) and the average amino acid identity (AAI) were obtained using the respective scripts of the enveomics collection (Rodriguez-R and Konstantinidis 2016). The percentage of conserved proteins (POCP) was calculated as described (Qin et al. 2014). The *rpoB* gene sequences were taken from publicly available genome annotations and sequence identities were determined as previously described (Bondoso et al. 2013). The alignment and matrix calculation were performed upon extracting a ca. 1298 bp region of the *rpoB* coding sequence that would have been sequenced with the described primer set. Alignment and matrix calculation were performed with Clustal Omega (Sievers and Higgins 2014).

### Light and electron microscopy

Cell morphological features, like size and shape, and cell division were observed under phase contrast microscopy or field emission scanning electron microscopy (FESEM). Phase contrast (Phaco) analyses were performed using a Nikon Eclipse Ti inverted microscope equipped with a Nikon DS-Ri2 camera (blue LED). Ten microliters of log phase-grown cells (in liquid medium) were immobilised in MatTek glass bottom dishes (35 mm, No. 1.5) employing a 1% (w/v) agarose cushion (Will et al. 2018). Images were analysed using the Nikon NIS-Elements software v.4.3. To determine the cell size, at least 100 representative cells were analysed manually (Annotations and Measurements, NIS-Elements). For FESEM, log phase-grown bacterial cells were fixed in 1% (v/v) formaldehyde in HEPES buffer (3 mM HEPES, 0.3 mM CaCl_2_, 0.3 mM MgCl_2_, 2.7 mM sucrose, pH 6.9) for 1 h on ice and washed with the same buffer (Rast et al. 2017). Fifty microliters of the fixed bacteria solution were placed on a poly-l-lysine cover slip and allowed to settle for 10 min. Cover slips were then fixed in 1% (v/v) glutaraldehyde in TE buffer (20 mM TRIS, 1 mM EDTA, pH 6.9) for 5 min. at room temperature and subsequently washed twice with TE buffer before dehydrating in a graded series of acetone (10, 30, 50, 70, 90, 100%, (v/v)) on ice for 10 min. at each concentration. Samples from the 100% acetone step were brought to room temperature before placing them in fresh 100% acetone. Samples were then subjected to critical point drying with liquid CO_2_ (CPD 300, Leica). Dried samples were covered with a gold/palladium (80/20) film by sputter coating (SCD 500, Bal-Tec) before examination in a field emission scanning electron microscope (Zeiss Merlin) using the Everhart Thornley HESE2 detector and the inlens SE detector in a 25:75 ratio at an acceleration voltage of 5 kV.

### Physiological analyses

For determination of the temperature optimum for growth, strain Pan189^T^ was cultivated in M1H NAG ASW medium at pH 7.5 at different temperatures ranging from 10 to 40 °C. The pH range (5.0, 6.0, 7.0, 7.5, 8.0, 9.0, 10.0) for growth was tested at 24 °C in M1H NAG ASW using different buffer systems. A concentration of 100 mM HEPES was used for cultivations at pH 7.0, 7.5 and 8.0. For the cultivation at pH 5.0 and 6.0 HEPES was replaced by 100 mM 2-(*N*-morpholino)ethanesulfonic acid (MES), whereas 100 mM *N*-cyclohexyl-2-aminoethanesulfonic acid (CHES) served as a buffering agent at pH 9.0 and 10.0. The requirement of vitamin B_12_ for growth of the strain was tested in liquid M1H NAG ASW medium with a modified vitamin solution lacking vitamin B_12_.

## Results and discussion

### BLAST analysis and phylogenetic inference

**Fig. 1:**
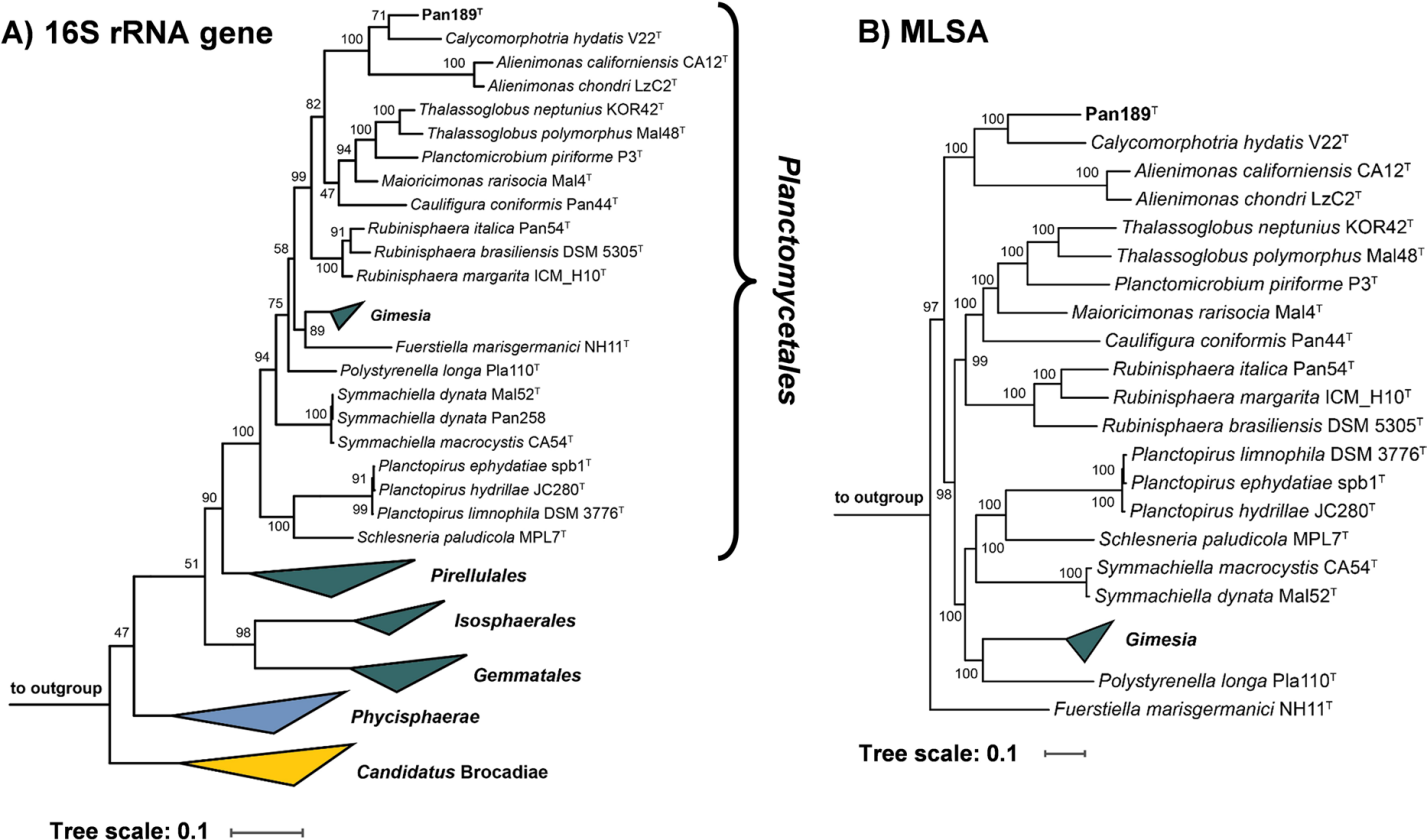
Maximum likelihood phylogenetic trees showing the position of strain Pan189^T^. **A** 16S rRNA gene sequence- and **B** MLSA-based phylogeny was computed as described in the Material and Methods section. Bootstrap values after 1,000 (16S rRNA gene sequence-based tree) and 500 re-samplings (MLSA-based tree) are given at the nodes (in %). Phylogenetic trees were visualized with iTOL v6. The scale bar indicates the number of substitutions per nucleotide position

**Fig. 2:**
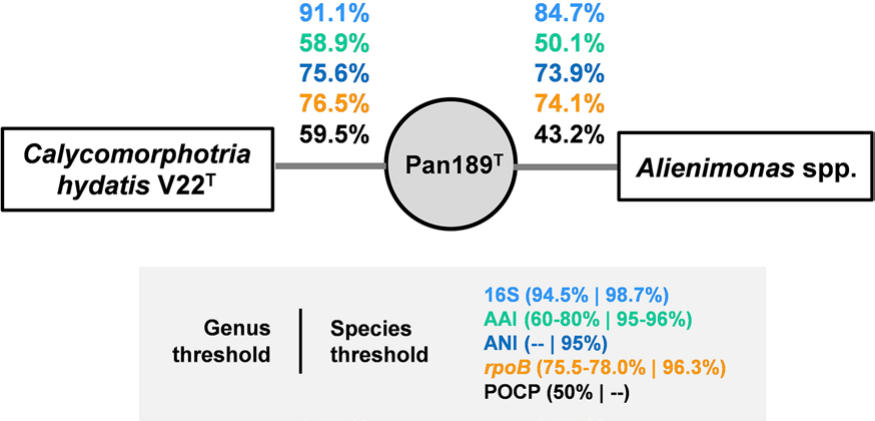
Comparison of phylogenetic markers for strain Pan189^T^ and its current closest neighbours. The numbers give the similarity values shared between strain Pan189^T^, *Calycomorphotria hydatis* V22^T^ and *Alienimonas* spp*.* (*A. californiensis* CA12^T^ and *A. chondri* LzC2^T^) for 16S rRNA gene sequence identity, identity of a 1298 bp partial sequence of the *rpoB* gene encoding the β-subunit of the RNA polymerase, average nucleotide identity (ANI), average amino acid identity (AAI), and percentage of conserved proteins (POCP)

To identify the current closest relatives of strain Pan189^T^, its 1521 bp 16S rRNA gene sequence was used for a BLAST analysis against the rRNA/ITS database at NCBI. The analysis places the novel isolate in the family *Planctomycetaceae* and identifies *Calycomorphotria hydatis* V22^T^ (Schubert et al. 2020) as the closest relative (16S rRNA gene sequence similarity of 91.1%). This finding is in line with the clustering of the strain in the 16S rRNA gene sequence- and MLSA-based phylogenetic trees (Figs. 1 and 2). The strain clustered on the same branch as *C. hydatis* V22^T^ and *Alienimonas* spp. (Boersma et al. 2020; Vitorino et al. 2020). The highest similarity value of the 16S rRNA gene sequence falls significantly below the genus threshold of 94.5% (Yarza et al. 2014), indicating that the novel isolate belongs to a novel species of a novel genus. According to the nucleotide blast analysis, the next uncultured environmental bacterial isolate has a 16S rRNA gene sequence identity of only 88.7%. This indicates that close relatives of the novel isolate have only rarely been observed in environmental samples from which 16S rRNA gene sequences have been deposited at NCBI.

Due to the clustering in both trees (Figs. 1 and 2), the strains *C. hydatis* V22^T^, *Alienimonas californiensis* CA12^T^ and *Alienimonas chondri* LzC2^T^ were used for comparison of the phenotypic and genomic features of strain Pan189^T^. The clustering of *Alienimonas* spp. with strain Pan189^T^ in the phylogenetic trees might be misleading as most of the phylogenetic marker values obtained during comparison of strain Pan189^T^ and *Alienimonas* spp. are lower compared to most of the other *Planctomycetaceae* members (data not shown). This is also reflected in the obtained branch lengths for *Alienimonas* spp. in both trees (Figs. 1 and 2).

The position of strain Pan189^T^ as a member of a novel species of a separate genus was substantiated by the analysis of other commonly used phylogenetic markers. All tested markers yielded *C. hydatis* V22^T^ as the current closest neighbour. The following values were obtained: 76.5% (*rpoB),* 58.9% (AAI) and 59.5% (POCP). The values for all analysed markers except POCP fell into or below the recommended genus threshold values/ranges of 75.5–78% (*rpoB*), 60–80% (AAI) and 50% (POCP) (Kallscheuer et al. 2020d; Luo et al. 2014; Qin et al. 2014; Rodriguez-R and Konstantinidis 2014). The POCP value for the comparison of strain Pan189^T^ and *C. hydatis* V22^T^ was relatively high and exceeded the 50% threshold value. A maximal ANI value of 75.6% ensures that the novel isolate does not belong to an already characterized species (species threshold 95%). Three out of four phylogenetic markers applicable for the delineation of genera support the conclusion of the introduction of a novel genus in case that this is also supported by differences in phenotypic and genomic characteristics of strain Pan189^T^.

### Analysis of genome-encoded features and pangenome construction

Strain Pan189^T^, *C. hydatis* V22^T^, *A. californiensis* CA12^T^ and *A. chondri* LzC2^T^ all have a genome size between 5.2 and 5.5 Mb. Hence, no large differences in the overall number of genes and protein-coding genes were found (Table 1). The same is true for the relative number of hypothetical protein-encoding genes, which falls between 25 and 30% (for an annotation with NCBI’s Prokaryotic Genome Annotation Pipeline). All four strains have the same number of rRNA genes (5S-16S-23S = 1-2-1), except for *A. californiensis* CA12^T^ which has one additional copy of the 5S and 23S rRNA genes. The most striking difference is the DNA G + C content. Strain Pan189^T^ and *C. hydatis* V22^T^ have a moderate DNA G + C content (54–59%), whereas the two *Alienimonas* species have a high DNA G + C content (68–71%) (Table 1).

**Table 1:** Phenotypic and genomic features of Pan189^T^ in comparison to *Calycomorphotria hydatis* V22^T^, *Alienimonas californiensis* CA12^T^ and *Alienimonas chondri* LzC2^T^

Characteristics	Pan189^T^	*C. hydatis* V22^T^	*A. californiensis* CA12^T^	*A. chondri* LzC2^T^
*Phenotypic features*				
Shape	Round grain rice-shaped	Round grain rice-shaped	Spherical to ovoid	Spherical to ovoid
Length (µm)	1.4 ± 0.2	1.6 ± 0.3	2.0 ± 0.2	1.4 ± 0.3
Width (µm)	0.7 ± 0.1	0.7 ± 0.2	1.5 ± 0.3	1.0 ± 0.2
Temperature range (optimum) (°C)	10–33 (24)	15–32 (30)	10–36 (27)	15–30
pH range (optimum)	7.0–8.0 (7.5)	6.0–10.0 (8.5)	6.0–9.0 (7.5)	5.5–10.0
Crateriform structures	Overall	Overall	Overall, except for the pole at which the flagellum is located	Uniform
Fimbriae	Overall	Few	Yes, polar	Yes
Stalk	opposite of budding pole	Short, opposite of budding pole	N.o.	N.o.
*Genomic features*				
Genome size [bp]	5,228,745	5,163,473	5,475,215	5,295,625
DNA G + C [%]	58.6	53.9	70.7	68.3
Coding density [%]	87.0	87.6	88.5	89.2
Completeness (Busco) [%]	96.4	96.3	95.9	95.5
Genes (total)	4,242	4,249	4,381	4,308
Genes per Mb	811	823	800	814
Protein-coding genes	4,159	4,143	4,303	4,139
Protein-coding genes per Mb	795	802	786	782
Hypothetical proteins	1,207	1,027	1,205	1,247
Hypothetical proteins [%]	29.0	24.8	28.0	30.1
rRNA genes (5S, 16S, 23S)	1, 2, 1	1, 2, 1	2, 2, 2	1, 2, 1
tRNAs	49	62	57	48
*Carbohydrate-active enzymes*				
Glycoside hydrolases	15	11	12	13
Glycosyl transferases	24	25	25	25
Polysaccharide lyase	0	2	1	1
Carbohydrate esterases	2	1	1	1
Carbohydrate-binding modules	3	3	1	1
*BGCs predicted by antiSMASH*				
Terpenoid	2	3	3	3
Type III PKS	1	0	1	1
Resorcinol	1	0	0	0
Other	1	1	1	1

N. o., not observed, Data for strains used for comparison was taken from the literature (Schubert et al. 2020; Boersma et al. 2020; Vitorino et al. 2020)

### Analysis of genome-encoded features and pangenome

Genes coding for enzymes participating in primary metabolism were analysed using the “Estimate Metabolism” tool of the anvi’o pipeline. This tool uses the KEGG module database to assign genes to central metabolic pathways in which the encoded enzymes are involved. The results show that catabolic and anabolic pathways of a typical heterotrophic bacterium are present in strain Pan189^T^. These include glycolysis (Embden-Meyerhof pathway), the tricarboxylic acid (TCA) cycle, gluconeogenesis, the pentose phosphate pathway as well as important anabolic pathways for fatty acids, amino acids, nucleotides and vitamins. The strain does not encode the required set of enzymes for de novo cobalamin (vitamin B_12_) biosynthesis. It harbours the cobalamin-dependent methionine synthase MetH but not the cobalamin-dependent class II ribonucleotide reductase NrdJ that is found in several members of the phylum. Instead of NrdJ, the cobalamin-independent heterodimeric class I enzyme NrdAB catalyzes the reduction of ribonucleotides to deoxyribonucleotides in strain Pan189^T^ and in the three strains *C. hydatis* V22^T^, *A. californiensis* CA12^T^ and *A. chondri* LzC2^T^ chosen for comparison.

The profiles of carbohydrate-active enzyme (CAZyme)- encoding genes and BGCs predicted by antiSMASH are similar between the four strains (Table 1). With a total number of 4–5 BGCs the genomes harbours ca. one BGC per Mb, which is in line with the predicted numbers of BGCs per Mb in the entire phylum (Kallscheuer and Jogler 2021). All four strains lack BGCs that typically comprise large genes, e.g., coding for type I polyketide synthases or non-ribosomal peptide synthetases. Instead, smaller genes (< 1500 bp) are present in BGCs putatively involved in the biosynthesis of terpenoids, resorcinol and type III polyketide synthase-derived compounds. Predicted CAZymes belong mostly to the classes of glycoside hydrolases and glycosyl transferases that together make up around 90% of the CAZyme portfolio of all four strains. Genes coding for putative polysaccharide lyases are exclusively absent in strain Pan189^T^ (Table 1). Taken together, lower numbers of CAZymes and BGCs putatively related to secondary metabolite production reflect the relatively small genomes of these strains compared to the other members of the class *Planctomycetia*.

**Fig. 3:**
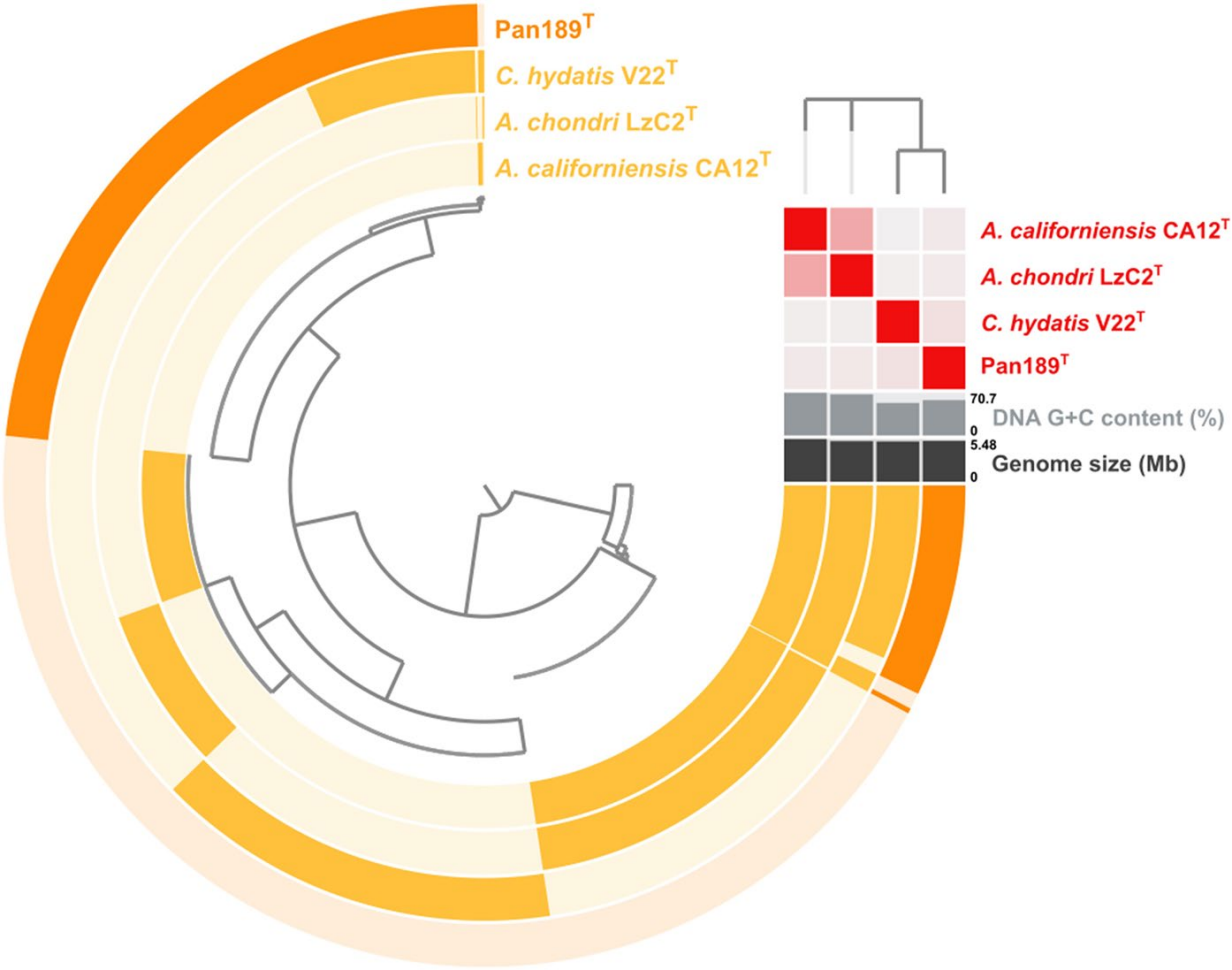
Pangenome of strain Pan189^T^ and its current closest neighbours. Each open circle represents the pangenome of all strains but is coloured darker when the gene is present in the respective genome. The tree in upper right corner reflects the relatedness of the strains based on the calculated average nucleotide identity (ANI)

To visualise the core and accessory genes in the genome of strain Pan189^T^, a pangenome was constructed based on the genomes of the novel isolate and the three strains that were used for comparison (Fig. 3). Although the strains have highly similar numbers of protein-coding genes, they only share a set of 892 genes with highly conserved sequences. The pangenome supports the results of the phylogenetic analysis since an additional number of 924 genes is shared by strain Pan189^T^ and its closer relative *C. hydatis* V22^T^, while the novel isolate only shares 117 genes exclusively with the two *Alienimonas* species. In strain  Pan189^T^, 2285 genes are singleton genes that are present in the strain but absent in any of the other strains included in the analysis. The construction of pangenomes is in general not only relevant for the comparison of phylogenetically closely related strains but contributes to identify genes that are important for cell biological processes that are so far not understood in members of the phylum, e.g., the asymmetric cell division or the suspected machinery for macromolecule uptake and degradation. Strains with relatively small genomes are of advantage for such bioinformatical analyses since narrowing down the numbers of candidate genes is easier than for strains with larger genomes and more accessory functions.

### Morphological and physiological analyses

**Fig. 4:**
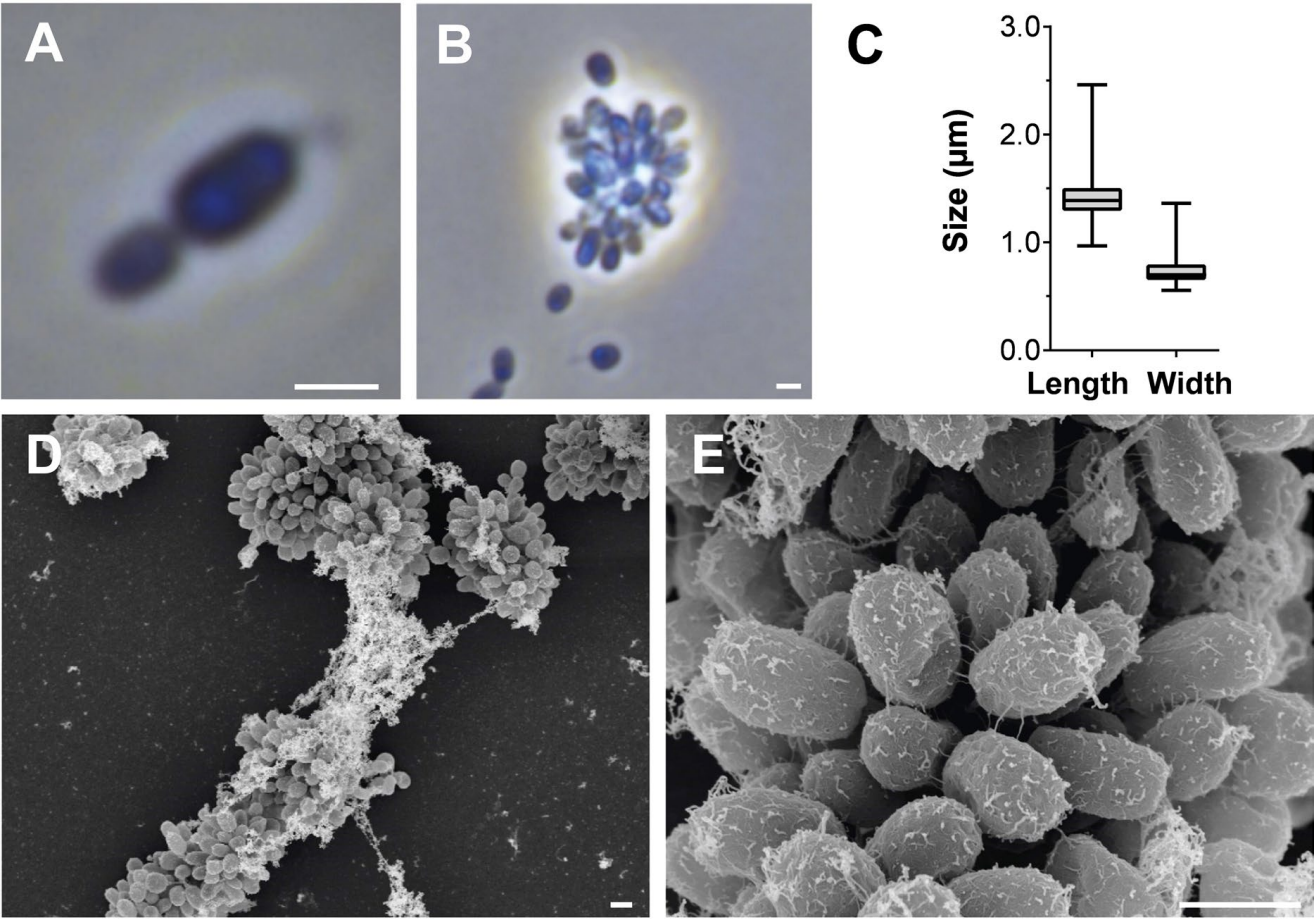
Microscopy images and cell size plot of strain Pan189^T^. Pictures from light microscopy (LM) (**A**, **B**), and scanning electron microscopy (SEM) (**D**, **E**) are shown. The scale bars indicate 1 µm. **C** Box plot of cell length and width based on 270 analysed cells

Basic features of strain Pan189^T^ comprising cell morphology, growth and mechanism of cell division are summarised in Table 1 and compared to *C. hydatis* V22^T^, *A. californiensis* CA12^T^ and *A. chondri* LzC2^T^. Phase contrast micrographs show that cells of strain Pan189^T^ are round grain rice-shaped (1.4 ± 0.2 × 0.7 ± 0.1 μm, Fig. 4A–C). Cells divide asymmetrically by polar budding and bear stalks opposite to the division pole. Daughter cells have the same shape as mother cells. SEM images show that cells can form strong aggregates. Crateriform structures and fimbriae are evenly distributed on the cell surface (Fig. 4D, E). The colonies of strain Pan189^T^ are pink-pigmented, which indicates the presence of carotenoids (Santana-Molina et al. 2022). Motile cells were present during the exponential phase, however, a dimorphic lifecycle was not observed (at least under laboratory-scale cultivation conditions).

**Fig. 5:**
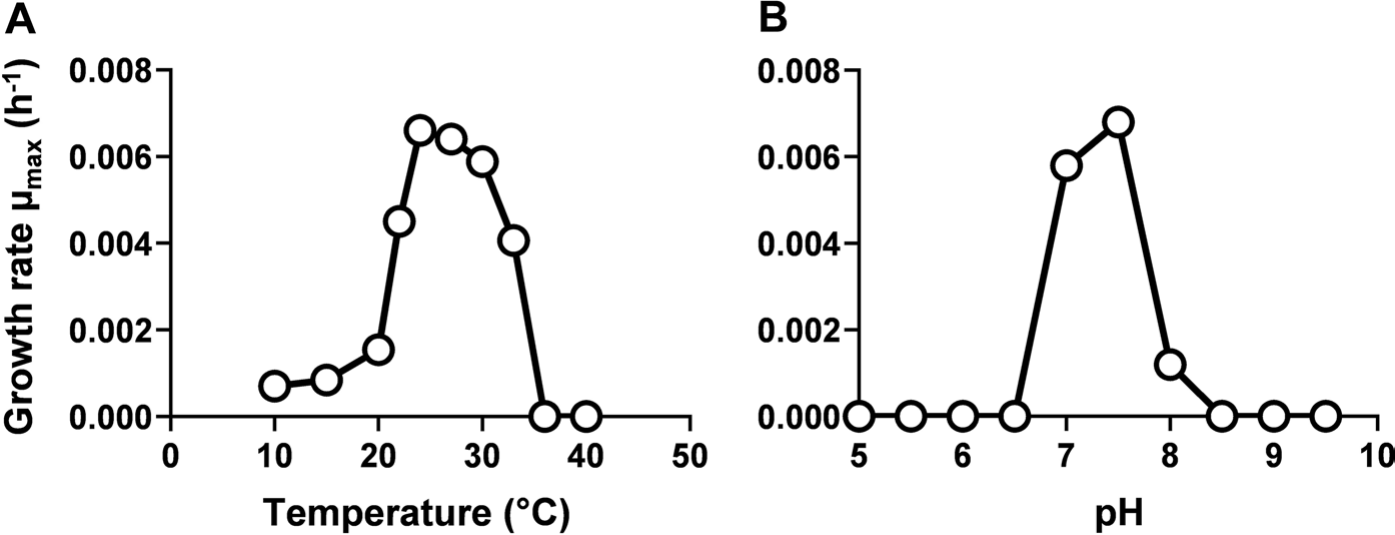
Temperature and pH optimum of the novel isolate. Data points show average growth rates obtained after cultivation in M1H NAG ASW medium in biological triplicates. Cultivations at different temperatures **A** were performed at pH 7.5. Cultivations at different pH values **B** were conducted at 24 °C

In M1H NAG ASW medium, strain Pan189^T^ was able to grow over a temperature range of 10–33 °C and a pH range of 7.0–8.0 (Fig. 5). The strain turned out to be aerobic, heterotrophic, mesophilic, and neutrophilic with optimal growth at 24 °C and pH 7.5. Cobalamin (vitamin B_12_) is not required for growth in the tested medium. This finding is in line with the presence of the cobalamin-independent ribonucleotide reductase in the strain. An auxotrophy for methionine in absence of supplemented cobalamin could not be tested due to the lack of a defined cultivation medium for this isolate. Even under the empirically determined optimal growth conditions, the strain only reached a maximal generation time of 4.3 days (102 h, µ_max_ = 0.0068 h^−1^) (Fig. 5). With this value, the strain belongs to the lower 5% in a ranking of growth speed of the class *Planctomycetia*. Typical growth rates observed for described members of the class fall between 0.001 and 0.01 h^−1^ (generation times of 7–70 h), although this number is highly biased when considering that faster-growing strains are more likely to be enriched and isolated from environmental samples using state-of-the-art isolation techniques. For morphological and physiological characteristics, only slight differences were observed between strain Pan189^T^ and *C. hydatis* V22^T^. More pronounced differences compared to the *Alienimonas* spp. may reflect the more distant relationship.

## Conclusion

Based on the phylogenetic distance and supported by phenotypic differences, strain Pan189^T^ should be delineated from the described genera in the family *Planctomycetaceae.* Hence, we introduce a novel genus and species, for which we propose the name *Stratiformator vulcanicus* gen. nov., sp. nov. Strain Pan189^T^ represents the type strain of the novel species.

### Description of *Stratiformator* gen. nov.

Stra.ti.for.ma’tor. L. neut. n. *stratum*, a layer, coat; L. masc. n. *formator*, a shaper, creator; N.L. masc. n. *Stratiformator* a bacterium that produces a biofilm.

Members of the genus have a Gram-negative cell envelope architecture and are aerobic heterotrophs with a mesophilic and neutrophilic growth profile. Cells are round grain rice-shaped and divide by polar budding. Crateriform structures are present. The DNA G + C content is around 59%. The genus belongs to the family *Planctomycetaceae*, order *Planctomycetales,* class *Planctomycetia,* phylum *Planctomycetota*. The type species of the genus is *Stratiformator vulcanicus*.

### Description of *Stratiformator vulcanicus* sp. nov.

vul.ca’ni.cus. L. masc. adj. *vulcanicus*, of or belonging to a volcano; corresponding to the origin of the strain from a volcano.

Round grain rice-shaped and pink-pigmented cells with an average size of 1.4 ± 0.2 × 0.7 ± 0.1 μm that form strong aggregates. Cells contain fimbriae and crateriform structures covering the entire cell surface and a stalk opposite of the division pole. The supplementation of vitamin B_12_ is not required for biomass formation in the tested medium. Growth of the type strain is observed over a range of 10–33 °C (optimum 24 °C) and at pH 7.0–8.0 (optimum 7.5). The type strain has a genome size of 5.2 Mb and a DNA G + C content of 58.6%. The type strain is Pan189^T^ (= DSM 101711^ T^ = CECT 30699^ T^). It was isolated from a red biofilm sampled close to the volcanic island Panarea in the Tyrrhenian Sea in September 2013.

## Data Availability

The GenBank/EMBL/DDBJ accession number for the 16S rRNA gene sequence of the strain Pan189^T^ is MK554509. The whole genome shotgun sequence for strain Pan189^T^ was deposited under GenBank/EMBL/DDBJ accession number CP036268.
